# Supplementary material for: SRSF3 Is a Critical Requirement for Inclusion of Exon 3 of BIS Pre-mRNA
Source: Cells. 2020 Oct 19;9(10):2325. doi: 10.3390/cells9102325 (PMC7589869; doi:10.3390/cells9102325)
Supplement: Supplementary file 1 [file cells-09-02325-s001.pdf]

Table S1. The sequence of siRNA used in this study

Targets	Sequence (5'-3')	Concentration (nM)
Negative Control	CCUACGCCACCAAUUUCGU	300
SRSF1	GCAGAUGAACUCGGGAUG	200
SRSF2	CCGCACUCGUUCUCGAUCUTT	200
SRSF3	GAGUGGAACUGUCGAAUGG	200
SRSF4	GGACUGCCUCCAAGUGGAATT	300
SRSF5	CCUCGAAAUGAUAGACGAATT	200
SRSF6	CGUUCUAGAUCUCGUUCAATT	200
SRSF7	CGACGUCCCUUUGAUCCAATT	200
SRSF8	GGAUUACAUUGGAGCCAAUTT	300
SRSF9	UGGUUAUGAUUAUGGCCAGTT	200
SRSF10	GGAUGUUCGUGAUGCUGAATT	200
SRSF11	GGAUACCUCUAGUAAAGAATT	200
SRSF12	GGUAGAACAGUAUAGGUAATT	300
hnRNP A1B2	GGAUUAUUUAAUAACAUUAT	100
hnRNP C	CGUCAGCGUGUAUCAGGAAT	100
hnRNP D	CAAUGUUGGUCUUAGUAAAT	100
hnRNP F	GCGUUCGUGCAGUUUGCCUT	100
hnRNP H	GGUAUAUUGAAAUCUUUAAT	200
hnRNP U	GUUUGUCUUGAUACUUAUAUU	100
hnRNP K	GGAAGUGACUUUGACUGCGAGUUGAUU	100
HuR	TGTGAAAGTGATCCGCGAC	100
SF3B2	CUGAUGUUGAGAUUGAGUAUU	100
SF3B4	CCGUAUCUUAUGCCUUCAA	200

Table S2. Primers and oligonucleotides used in this study

Used for	Targets		Sequences (5'-3')
qRT-PCR	hSRSF1	Forward	CAGAGTGGTTGTCTCTG
		Reverse	CTCCACGACACCAGTGCC
	hSRSF2	Forward	GGACGCCGGAGCCGCAG
		Reverse	GAGATCGAGAACGAGTGC
	hSRSF3	Forward	ATGCATCGTGATTCCTG
		Reverse	CTGCGACGAGGTGGAGG
	hSRSF4	Forward	GTTACGGTTCTGGACGC
		Reverse	GCTCCGGGAGCGGGAG
	hSRSF5	Forward	GATCCAAGGGATGCAGATG
		Reverse	CTATCATTTCGAGGTCTGCG
	hSRSF6	Forward	GTGGATACAGCAGTCGG
		Reverse	CTGGATCTGCTTCCAGAG
	hSRSF7	Forward	GGTCTAGATCACATTCTCG
		Reverse	CCAGACCTAGATCTTCTG
	hSRSF8	Forward	ATGAGGACAGGTGGCCG
		Reverse	GCGCTGCACTGGGCGCTG
	hSRSF9	Forward	GGCCAGTGTCGGCTTCGTG
		Reverse	GGTGTCATCCAGTTTACGC
	hSRSF10	Forward	GTCTGA AGACTTGCGGCG
		Reverse	CCGACTTCTTGATCTCCTCC
	hSRSF11	Forward	CAGGAGCGAGAACCCGAG
		Reverse	CTTCTGCATATGGTACGAC
	hSRSF12	Forward	GCCTGAGGACTTGCGCCG
		Reverse	AATCTGACGGCCACATACC
	hHnRNP A1B2	Forward	AGCTTTGAAACCACAGAAGAA
		Reverse	TTGATCTTTTGCTTGCAGGA
	hHnRNP C	Forward	GTTACCCAGCACGTGTACC
		Reverse	GGCCTGAAGGTCATCTCC
	hHnRNP D	Forward	TGGGAAGGTGATTGATCC
		Reverse	AAGCAGAACCCACGCCTC
	hHnRNP F	Forward	ACTGCCAGGAGGTACATTGG
		Reverse	CTGAGGTCTCTCCCGAACAG
	hHnRNP H	Forward	GTGCAGTTTGCTTCACAGGA
		Reverse	CCCCAGGTCTGTCATAAGGA
	hHnRNP U	Forward	AGCCAAATCTCCTCAGCC
		Reverse	TGACCAGCCAATACGAAC
	hHnRNP K	Forward	GAAGAAACCTTCCCTAACACTGAA
		Reverse	TCAGAATTTCTCCAATTGTTTCAA
	hHuR	Forward	AACTACGTGACCGCGAAGG
		Reverse	CGCCCAAACCGAGAGAACA
	hSF3B2	Forward	AAGTGATGAAGACAAACCAGATGA
		Reverse	CATAACCGTGGACATGTCATAAAT
	hSF3B4	Forward	AGTCGACAATGGCTGCCGGGCCGATCT
		Reverse	CCTCGAGGAAGGAAAATGTGAATTTA
	hβ-Actin	Forward	AGTACTCCGTGTGGATCGGC
		Reverse	GCTGATCCACATCTGCTGGA
RT-PCR	BIS Exon1	Forward	CTTCGTGGACCACAACAGC

	hBIS Exon4	Reverse	ACAGGTTTAGAATCCACCTCTTTG
	mBIS Exon4	Reverse	TCTGCCTCCCTTCGGATCACT
	GFP	Forward	GCATGGACGAGCTGTACAAG
	hGAPDH	Forward<br>Reverse	CCATGTTCGTCATGGGTGTGAACCA<br>GCCAGTAGAGGCAGGGATGATGTTC
	mGAPDH	Forward<br>Reverse	GGTGTGAACGGATTTGGCCGTATT<br>GGCCTTGACTGTGCCGTTGAATTT
Biotin pull down	Exon 1	Forward	CCAAGCTTCTAATACGACTCACTATAGGGAG AATGAGCGCCGCCACCCACTC
		Reverse	CTTGGGGCCCTCAGAGGGCA
	Exon 2	Forward	CCAAGCTTCTAATACGACTCACTATAGGGAG AGAGACTCCATCCTCTGCCAATGGC
		Reverse	CTCAGGTCCGTGGGAGGCTG
	Exon 3	Forward	CCAAGCTTCTAATACGACTCACTATAGGGAG ACGGTCCCAGTCTCCAGCTGCC
		Reverse	CTGAGGCCTGTCGACCACGG
	Exon 4	Forward	CCAAGCTTCTAATACGACTCACTATAGGGAG ACAGCCCATGACCCATCGAGAAACT
		Reverse	CTACGGTGCTGCTGGGTTACCAGG
	Exon 3-a	Oligo	CCAAGCTTCTAATACGACTCACTATAGGGAG ACGGTCCCAGTCTCCAGCTGCCTCTGACTG CTCATCCTCATCCTCCTCGGCCAGCCTGCCT TCCTCCGGCAGGAGCAGCCT
	Exon 3-b	Oligo	CCAAGCTTCTAATACGACTCACTATAGGGAG AGGGCAGTCACCAGCTCCCGCGGGGGTACA TCTCCATTCCGGTGATACACGAGCAGAACGT TACCCGGCCAGCAGCCCAGC
	Exon 3-c	Oligo	CCAAGCTTCTAATACGACTCACTATAGGGAG ACCTGTGTACCACAAGATCCAGGGGGATGA CTGGGAGCCCCGGCCCCTGCGGGCGGCAT CCCCGTTCAGGTCATCTGTCCA
	Exon 3-d	Oligo	CCAAGCTTCTAATACGACTCACTATAGGGAG AGGGTGCATCGAGCCGGGAGGGCTCACCA GCCAGGAGCAGCACGCCACTCCACTCCCCC TCGCCCATCCGTGTGCACACCG
	Exon 3-a-Mut	Oligo	CCAAGCTTCTAATACGACTCACTATAGGGAG ACGGTCCCAGTCTCCAGCTGCCTCTGACTC CTCGGCCAGCCTGCCTTCCTCCGGCAGGAG CAGCCTGGGCAGTCACCAGCT
GFP-BIS WT Minigene Cloning	BIS-E2	Forward	TCTCGAGCTGAGACTCCATCCTCTGCC AAT
	BIS-I3	Reverse	CGAATTCAGGGAGAACCTATACTGTGC
	BIS-I3	Forward	TGAATTCCTGGGCTCAAGCGATTCT
	BIS-E4	Reverse	CGAATTCTCTGAAATGCATGCAACTTA
GFP-BIS Mut Minigene Cloning	ΔE3a	Forward	CTCTGACTGCCTCCTCGGCCAGCCTGCCT
		Reverse	GGCCGAGGAGGCAGTCAGAGGCAGCTGGA
BIS cloning	BIS E1<br>BIS E4	Forward	GCTCGAGACATGAGCGCCGCCACCCAC
		Reverse	GGAATTCCTACGGTGCTGCTGGGTTACCAG

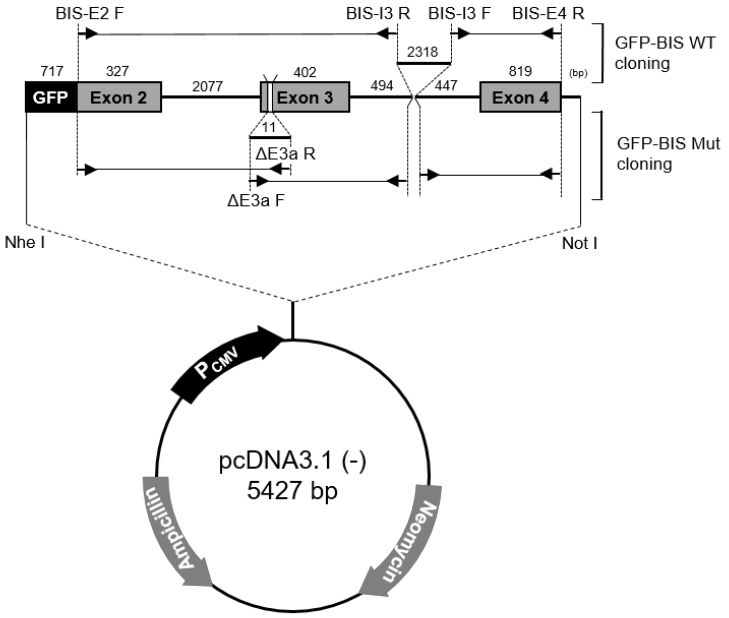

Figure S1: The simple map for minigene and the primers used for cloning.
The GFP-BIS wild type (WT) minigene was prepared by two PCR steps. First, using BIS-E2F and BIS-I3R primers, the fragment including exon 2, exon 3, and the indicated introns was amplified and ligated into the pEGEF-C1 vector. The second fragment including exon 4 and the indicated introns was amplified using BIS-I3F and BIS-E4R primers and ligated into the pJET1.2/blunt vector. The first fragment including GFP sequence and the second fragment were inserted into and pcDNA3.1(-) vector at Nhe I and EcoR I, and EcoR I and Not I, respectively. GFP-BIS deletion mutant (Mut) vectors were prepared by two primer sets, BIS-E2F and ΔE3a R, and ΔE3a F and BIS-I3R, for the first fragment. The sequences of each primers are listed in Table S2.

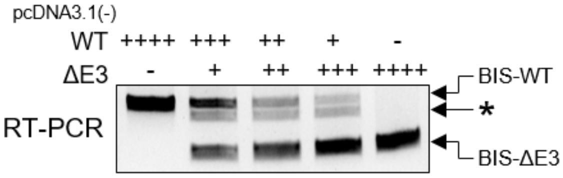

Figure S2: The generation of the artifact band during RT-PCR from two templates, wild type (BIS-WT) and isoform deleted in exon 3 (BIS-ΔE3).
To verify the generation of the artifact band, which is smaller than WT-BIS (indicated as asterisk), PCR was performed using two kinds of vectors, BIS-WT and BIS-ΔE3 in pcDNA3.1(-), simultaneously, with several combinations in the amounts of each templates. The primer sets on exon 1 and exon 4 were used as in Figure 1A. Note that the artifact only appeared when the two templates with different lengths were present.

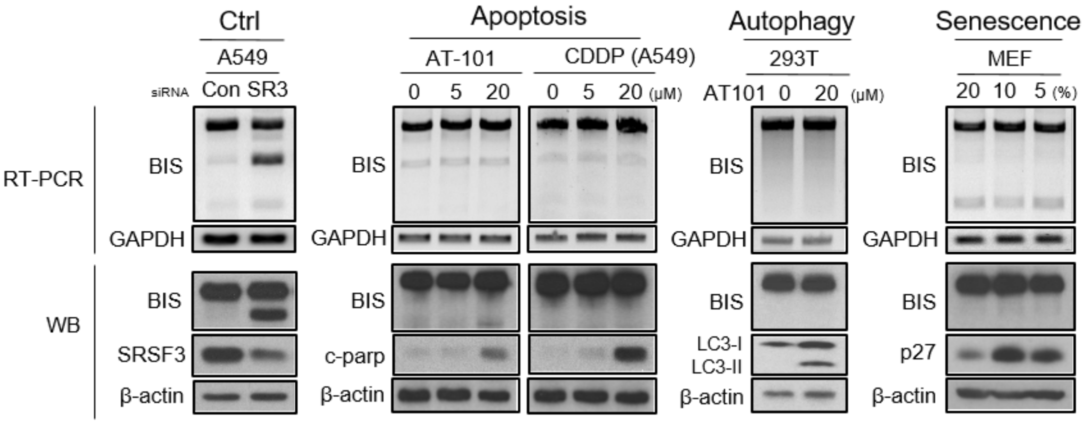

Figure S3: The expression pattern of BIS mRNA and protein levels under various cellular processes.
To compare BIS-WT or BIS-ΔE3 expression, A549 cells were treated with 100 nM of control siRNA or SRSF3 siRNA for 24 h (Ctrl). For induction of apoptosis, A549 cells were treated with 0, 5, or 20 μM of AT-101, pan Bcl-2 inhibitor, and DNA replication inhibitor CDDP drugs for 24 h. The cleaved-PARP (c-parp) was detected as a marker of apoptosis. For induction of autophagy, 293T cells were treated with 20 μM of AT-101 for 48 h. The increase of LC3-II was examined as a marker of autophagy induction. In addition, the early passage of mouse embryonic fibroblast (MEF) cells were cultured in DMEM supplemented with 20, 10, 5% (v/v) FBS for 24 h and p27 was used as a marker of senescence. Note that a significant increase in the deletion of exon 3 of BIS pre-mRNA was not observed in these conditions.

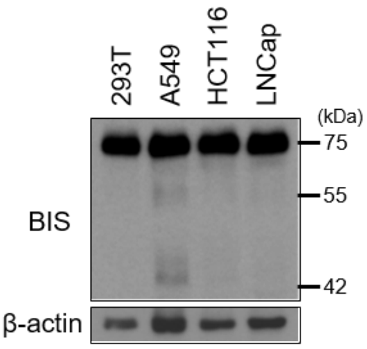

Figure S4: The BIS expression in several human tumor cell lines.
Western blotting for BIS expression patterns indicated that the truncated BIS levels were very low in 293T human embryonic kidney cells, A549 non-small cell lung cancer cells, HCT116 colorectal carcinoma cells, and LNCap prostate adenocarcinoma cells.
